# Piloting data linkage in a prospective cohort study of a GP referral scheme to avoid unnecessary emergency department conveyance

**DOI:** 10.1186/s12873-020-00343-w

**Published:** 2020-06-12

**Authors:** Joanna M. Blodgett, Duncan J. Robertson, David Ratcliffe, Kenneth Rockwood

**Affiliations:** 1 MRC Unit for Lifelong Health and Ageing at UCL, 1-19 Torrington Place, London, WC1E 7HB UK; 2 North West Ambulance Service, NHS Trust, Bolton, Greater Manchester, UK; 3 Welsh Ambulance Services Trust, Saint Asaph, Denbighshire UK; 4 Greater Manchester Health and Social Care Partnership, Greater Manchester, UK; 5 Salford Royal NHS Foundation Trust, Greater Manchester, UK; 6 Division of Geriatric Medicine, Department of Medicine, Dalhousie University, Halifax, N.S Canada

**Keywords:** Emergency care, Ambulance, Non-conveyance, Alternate route of care

## Abstract

**Background:**

UK Ambulance services are under pressure to safely stream appropriate patients away from the Emergency Department (ED). Even so, there has been little evaluation of patient outcomes. We investigated differences between patients who are conveyed directly to ED after calling 999 and those referred by an ambulance crew to a novel GP referral scheme.

**Methods:**

This was a prospective study comparing patients from two cohorts, one conveyed directly to the ED (*n* = 4219) and the other referred to a GP by the on-scene paramedic (*n* = 321). To compare differences in patient outcomes, we include follow-up data of a smaller subset of each cohort (up to *n* = 150 in each) including hospital admission, history of long-term illness, previous ED attendance, length of stay, hospital investigations, internal transfers, 30-day re-admission and 10-month mortality.

**Results:**

Older individuals, females, and those with minor incidents were more likely to be referred to a GP than conveyed directly to ED. Of those patients referred to the GP, only 22.4% presented at ED within 30 days. These patients were more likely to be admitted then than were those initially conveyed directly to ED (59% vs 31%). Those conveyed to ED had a higher risk of death compared to those who were referred to the GP (HR: 2.59; 95% CI 1.14–5.89), however when analyses were restricted to those who presented at ED within 30 days, there was no difference in mortality risk (HR: 1.45; 95% CI 0.58–3.65).

**Conclusions:**

Despite limited data and a small sample size, there were differences between patients conveyed directly to ED and those who were referred into GP care. Initial evidence suggests that referring individuals to a GP may provide an appropriate and safe alternative path of care. This pilot study demonstrated a need for larger scale, methodologically rigorous study to demonstrate the benefits of alternative conveyance schemes and recommend changes to the current system of urgent and emergency care.

## Background

As the number of 999 calls and the number of patients who present at Emergency Departments (EDs) increase each year [1], UK Ambulance services are under pressure to safely stream patients to alternative routes of care [2, 3]. Hospitalisation for acute medical illness can frequently trigger short and long-term disability [4, 5]. Prognosis for recovery from such disability is poor; as few as 30% of patients return to their pre-hospitalized level of self-care functioning after 12 months [6]. The Department of Health acknowledged that unnecessary hospital attendances and longer stays can lead to worse health outcomes and increased long-term care needs of older adults [7] while contributing to the steadily rising costs for patients, Clinical Commissioning Groups, social care providers and the National Health Service (NHS) [8].

UK ambulance trusts have introduced schemes aimed at decreasing rates of conveyance to EDs by streaming patients to alternative treatment or referral options based on clinical acuity [3]. Although some trusts have formal mechanisms for referral, others leave the paramedic to make discretionary decisions [9]. A multidisciplinary clinical group at the North West Ambulance Service (NWAS) developed a triage protocol, Paramedic Pathfinder, to categorise patients into: emergency care, urgent and community supported care or self-care [10]. This system aims to reduce the number of unnecessary ED visits by directing paramedics to safe triage decisions that allow alternatives including urgent care centres and referral into primary care.

Patients who are triaged into the urgent and community supported care pathway can be referred into the General Practitioner (GP) Acute Visiting Scheme (AVS). This innovative program enables a structured and governed referral to partner GP providers, avoiding unnecessary ED admission by providing a clinical safety net. The safety net is provided by a clinician to clinician handover between the attending paramedic and the GP, in collaboration with the wishes of the patient.

In the 2015–2016 fiscal year, there were 1.15 million 999 calls leading to 1.03 million incidents attended to by NWAS paramedics. Paramedics attempted 66,836 referrals (~ 5%) to the GP AVS, of which 90% were accepted [3]; without the scheme, these patients would have been conveyed directly to ED or referred on an ad hoc basis without assurances. Qualitative studies have demonstrated both the paramedics and GPs’ commitment to and belief in the scheme, and that Paramedic Pathfinder can successfully stream patients away from the ED. [11, 12] Although much evidence suggests early success [3, 10–12], there has been no formal enquiry to explore the efficacy or effectiveness of such referrals. A lack of follow-up evidence has resulted in a knowledge gap regarding the safety and health outcomes of patients referred to the scheme [3].

The aim of this pilot study is to determine feasibility of systematically assessing differences between patients who are conveyed directly to ED compared with those who are referred by an ambulance crew to the GP AVS referral scheme. As no previous study has examined the efficacy of such schemes, or the safety of their patients, we aimed to test the methodology on a small-scale to determine whether further inquiry into the benefits and risks is needed and feasible.

## Methods

### Setting, sample and data linkage

The North West Ambulance Service NHS Trust provides urgent and emergency care services to a population of 7 million people in Cumbria, Lancashire, Greater Manchester, Merseyside and Cheshire (North West England). The City of Salford is a Metropolitan borough in Greater Manchester (2011 census, population: 233900 [13]) and is situated directly west of Manchester city centre. Patients in the Salford area attended by NWAS are typically conveyed to the nearest ED, the Salford Royal NHS Foundation Trust hospital.

Between June 1st 2015 and August 31st 2015, 5283 patients aged ≥17 in the Salford area attended to by an on-scene NWAS ambulance crew. Patients with mental health related incidents and those who were referred to the ambulance by a healthcare practitioner were excluded (*n* = 743). Cohort 1 consisted of 4219 patients who were conveyed directly to the Salford Royal Hospital ED. Cohort 2 consisted of 321 patients who were referred and accepted into the NWAS GP referral scheme. A smaller subsample of each cohort was followed up by linking patient’s ambulance data from NWAS to ED data and GP referral data; these smaller samples aimed to test the feasibility and information governance processes. A probabilistic linkage strategy based on last name, initial, age and sex was used to link hospital and GP referral data. Each subsample was a convenience sample of the first 150 patients occurring from June 1st onwards. Patients who could not be traced due to missing data were excluded (13/150 in sub-cohort 1; 7/150 in sub-cohort 2).

### Patient data

Data obtained from the ambulance, GP referral and hospital systems were linked. For all individuals (*n* = 5283), *age*, *sex* and *call description* were obtained from the Computer Aided Dispatch System (CADS) via the NWAS Informatics team. Within CADS, the Medical Priority Dispatch System (MPDS) uses standardised protocols to triage patients at the point of call, allowing appropriate aid to be dispatched to medical emergencies [14]. This computerised triage system assigns codes to provide the *call description* and indicate the specific complaints (e.g. 1- Abdominal pain/problems, 2- Allergies/Envenomation, etc.).

Data from GP referral and hospital services enabled identification of all patients who were accepted by the GP and who were admitted to the hospital within 30 days of the ambulance- attended episode. Linked hospital Electronic Patient Records (EPR) provided *history of long term illness* (any EPR medical history), *history of previous ED attendances* (in last 3 years), *length of hospital stay* (days), *hospital investigations* (count: 0–14), *internal transfers between wards* (count: 0–6), *30-day re-admission* (yes/no) and *mortality status* (date of death; 10 month follow-up from date of ambulance incident). Possible *hospital investigations* included: biochemistry, blood cultures, blood glucose test, cardiac enzymes test, clotting screen test, computerised tomography, cross match blood test, electrocardiogram, haematology, magnetic resonance imaging, radiology, toxicology, urinalysis and urine cultures.

### Ethical approval

This pilot study was a service evaluation not requiring formal NHS ethical approval; the study received ethical approval guidance from the Salford Royal NHS Foundation Trust (SRFT) information governance team, underwent discussions with NHS Ethics and was logged with the NWAS Research and Development Board.

### Statistical analyses

Differences in age, sex and call description of those conveyed directly to ED and those referred to the GP were first compared in the full cohort and then repeated in the sub-cohorts (e.g. those linked to hospital and/or GP data); t-tests were used to compare mean age, chi square tests were used to compare proportion of men and women and a Bonferroni post hoc test was to identify differences in call description codes. After linkage of hospital data, sex and age-adjusted logistic regressions were used to examine the association between pathway (e.g. direct AE conveyance vs GP referral) and 30-day hospital admission (yes/no). Associations were first examined in the full sub-cohort sample and then, in a restricted sample of those who presented at ED within 30 days.

Next, chi square tests and t-tests were used to compare history of long term illnesses, frequency of previous ED attendances, length of stay, number of investigations and number of ward transfers between those admitted following direct ED conveyance and those admitted after referral to the GP scheme. Finally, sex and age-adjusted Cox regressions examined associations with 10-month mortality in all sub-cohorts; as above, associations were examined in the full sub-cohort sample and then in a restricted sample of those who presented at ED. Individuals who were still alive were censored at 300 days. The level of significance for all analyses was set at 0.05. All analyses were calculated using IBM SPSS Statistics, version 22.0.

## Results

### Full cohort differences

**Fig. 1:**
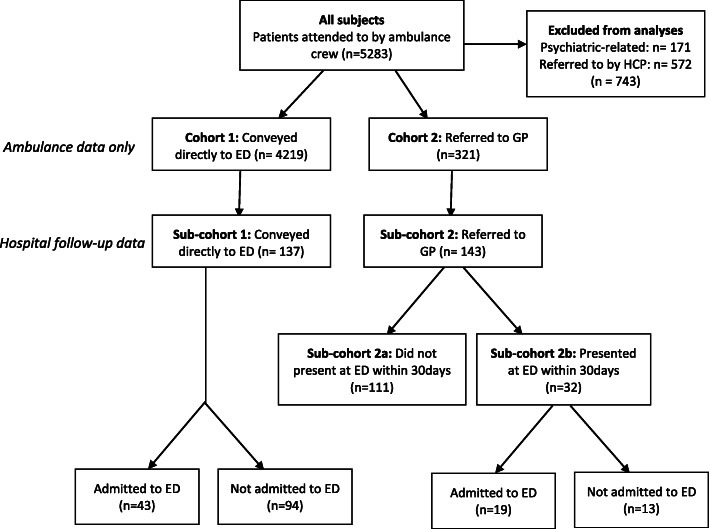
Flow diagram demonstrating patient pathway by cohort (and/or sub-cohorts) after being attended on scene by an ambulance crew

Of the 5283 patients who were attended on-scene by a paramedic between June 1st 2015 and August 31st 2015, 171 were excluded as mental health-related incidents and 572 were excluded as they had been initially referred to emergency services by a healthcare practitioner (HCP). Of the remainder 4540, 4219 (92.9%) were conveyed directly to the ED and 321 (7.1%) were referred to a GP (see Fig. 1). Those who were referred to the GP scheme were older and more often female (64.2 ± 23.4 years old; 58.6% female) than those conveyed directly to the ED (57.6 ± 22.7; 51.1% female) (*p* < 0.001 for age and *p* < 0.01 for sex; see Table 1). Patients were significantly more likely to be conveyed directly to ED if they were coded by the computerised triage process at the point of call as traumatic injuries (3.0% vs 0.3%), falls (10.4% vs 6.2%), convulsions/fitting (4.5% vs 0.6%) or overdose/poisoning (3.2% vs 1.2%; all *p* < 0.01). Conversely, incidents were more likely to be referred to GP if they were coded as an NHS 111 electronic transfer (27.4% vs 14.6%), a “sick person” (9.7% vs 5.5%), non-traumatic back pain (1.2% vs 0.4%) or diabetic problems (1.6% vs 0.5%; all *p* < 0.01).

**Table 1:** Characteristics of sample of patients seen after 999 calls: full cohort and sub-cohort follow up

	Cohort 1: Conveyed to ED	Cohort 2: Referred to GP	***p***-value
**Full cohort**			
Sample size (n)	4219	321	–
Mean age ± SD (range)	57.6 ± 22.7 (17–102)	64.2 ± 23.4 (17–102)	< 0.001
Female (n, %)	2156 (51.1%)	188 (58.6%)	< 0.01
**Sub-cohorts (those followed up)**			
Sample size (n)	137	143	–
Mean age ± SD (range)	58.4 ± 22.7(18–102)	63.1 ± 24.1(18–98)	0.10
Female (n, %)	80 (58.4%)	81 (56.6%)	0.77

### Follow-up data in sub-cohorts

There were no sex or age differences between those conveyed directly to ED and those referred to GP (see Table 1, sub-cohorts). Of the 143 patients referred to the GP scheme, 32 (22.4%) went to the ED within 30 days, indicating that 77.6% of patients were successfully diverted away from ED (e.g. 30-day deflection rate). Of those from the GP scheme, there was no difference in age (*p* = 0.10), sex (*p* = 0.72) or call description (*p* = 0.84) between those who presented within 30 days and those who did not. Of the 32 patients who presented at ED after referral to a GP, 19 (59.4%; 13.3% of original 143) were admitted to the hospital, while 43/137 patients (31.4%) were admitted upon their direct conveyance to ED (see Fig. 1, Table 2).

**Table 2:** Characteristics of those conveyed directly to ED and those referred to GP (subsample)

	Sub-cohort 1: Conveyed to ED (***n*** = 137)	Sub-cohort 2: Referred to GP (***n*** = 143)	***p***-value
**Sub-cohorts (those followed-up)**			
Conveyed directly to ED (n, %)	137 (100%)	–	–
Presented at ED within 30 days of ambulance call (n, %)	–	32 (22.4%)	–
Admitted (n, %)	43 (31.4%)	19 (13.3%; 59.4%) ^a^	–^b^
**Patients admitted to ED**	*n* = 43	*n* = 19	
*History*			
Long term illness (n, %)	5 (3.6%)	6 (4.2%; 18.8%) ^a^	< 0.005
Previous ED attendances (mean ± SD)	11.4 ± 29.3	11.9 ± 21.6	0.92
*Outcomes*			
Length of stay (mean ± SD)	8.3 ± 16.0	7.4 ± 9.3	0.81
Number of hospital investigations (mean ± SD)	4.7 ± 1.8	4.8 ± 1.4	0.59
Number of internal transfers (mean ± SD)	2.0 ± 0.9	2.40 ± 1.1	0.31
30-day re-admission (n, %)	8 (18.6%)	5 (26.3%)	0.49
10-month mortality (n,%)	16 (11.7%)	9 (6.3%; 28.1%)^a^	–^a^

^a^ proportion (%) of sub-cohort (*n* = 143) and of those presenting at ED (*n* = 32)

^b^ see linear regression and Cox-regression in text

Those who were directly conveyed to ED (sub-cohort 1: *n* = 137) were 3.52 (95% CI: 1.87, 6.62) times more likely to be admitted than those who were originally referred to GP (sub-cohort 2: *n* = 143). However, a restricted analysis of those who presented at ED suggested that those who later presented to the ED after having been referred to GP (*n* = 32) were more likely to be admitted than those directly conveyed (*n* = 137) (OR: 2.72; 95% CI 1.20–6.20). There was no difference between pathway groups in the number of previous ED attendances (*p* = 0.92), length of stay (0.81), number of investigations in hospital (0.59), number of transfers between wards (*p* = 0.31) or 30-day re-admission (*p* = 0.49) (Table 2), however those referred to a GP were more likely to have a history of a long-term illness (18.8% vs 3.6%; *p* < 0.005).

In an age and sex-adjusted Cox regression model of both sub-cohorts (*n* = 280), those directly conveyed to ED had a 2.59 (95% CI 1.14–5.89) times higher risk of death compared to those who were referred to a GP. However, in the restricted analyses of those who presented at ED only (*n* = 169), there was no difference in mortality risk between pathways (HR: 1.45; 95% CI 0.58–3.65).

## Discussion

This study of patients who called 999 demonstrated differences between those who were conveyed directly to ED by an ambulance and those who were referred directly to a GP via the NWAS GP Acute Visiting Scheme. The GP scheme was able to successfully stream away from ED; one in four referred patients (22.4%) attended ED within 30 days and one in eight (13.3%) required hospitalization. Despite limited data and a small sample size, there were differences between cohorts in age and sex characteristics, admission rates, mortality, and history of long-term illness. This pilot demonstrates a need for a large-scale investigation of patient safety as well as formal economic analysis to determine the costs of the scheme and any potential system-wide savings that non-ED conveyance can bring.

Older women with call descriptions for less severe or acute problems were more likely to be referred to a GP. This is consistent with reports that over 50% of referrals are elderly medical patients [15]. These older patients often have multiple co-morbidities [16] that can complicate the paramedic’s assessment [17]. One important aspect of the paramedic’s assessment is to separate chronic problems from issues requiring immediate care. For example, consider Subject A, an otherwise healthy patient presenting with severe breathing problems, and Subject B, a chronic smoker who suffers with COPD. Subject A may need to be conveyed directly to ED for treatment, whereas Subject B’s condition may be better treated via a GP in the community. Unsurprisingly, patients who were conveyed directly to ED had more urgent, severe causes of complaint. Despite a small sample size, those with less severe call descriptions - “sick person”, non-traumatic back pain, diabetic-related complaints or if the call was transferred from the non-emergency NHS 111 line - were more likely to be referred to GP. This is consistent with lower severity of illness/injury in those who do not require immediate conveyance and suggests accurate use of Paramedic Pathfinder [9] by the paramedic at this stage of the care path model.

There were, however, no differences in hospital data between those conveyed directly to ED and those who presented at ED within 30 days of being referred to GP; this included previous ED attendances, length of stay, number of investigations, number of transfers and 30-day re-admission. The finding that those referred to GP were more likely to have a long-term condition supports paramedics’ ability to effectively separate a non-emergency chronic condition from a situation requiring immediate care. Patients conveyed directly to ED had a higher risk of death than those referred directly to the GP scheme [HR = 2.59 (1.14–5.89)], however there was no significant difference in 10-month mortality risk between those conveyed directly and those who presented at ED within 30 days of GP referral [HR = 1.45; 95% CI 0.58–3.65]. The higher risk of death in those conveyed directly to ED clearly reflects the higher severity of call (requiring immediate ED conveyance), which may explain early mortality. Conversely, the non-significant mortality risk difference between those who those conveyed directly to ED and those who presented at ED within 30 days may indicate that the GP referral scheme is able to successfully identify and deflect those who do not require immediate care.

One of the most important differences between groups was the lower admittance rate of those directly conveyed to ED (31.4%) compared to those who presented at ED after being referred to GP (59.4%). The lower rate of admission after direct ED conveyance may suggest that many patients who were conveyed directly did not require emergency care and could have received adequate care in a different setting. Community paramedicine initiatives have emerged worldwide to tackle this challenge, where specially trained paramedics are trained to use their clinical skills to keep patients out of the emergency department [18]. A 100% successful deflection rate is neither possible nor desirable, as it may indicate an algorithm that lacks sensitivity. Even so, the high deflection rate here indicates scheme success as these patients may have otherwise been conveyed directly to ED without the availability of the alternative GP referral route, or referred using a less robust system. This is a higher deflection rate than has been noted before [3]. It is possible that patients who were referred to GP presented at an alternate ED department than SRFT within the 30 days; although this would be unusual as the patients reside in the Salford area. However, the deflection rate reported here may underestimate the success of the GP referral scheme; this is because future presentation at ED may have been for an incident or condition unrelated to the original ambulance incident.

This pilot study has several limitations and as such the results must be interpreted with caution. The availability of ambulance and hospital data restricted what comparisons could made between groups. As the ambulance data collected are for clinical purposes only, limited ambulance data could be used to statistically examine cohort differences. Furthermore, the call description is coded automatically by the computerized triage process and the exact reason for the call or the conveyance is not always clear; for example, a fall could be coded under falls or it could be coded under local codes “NHS 111 transfer” as they are both secondary triage outcomes. As ambulance systems move to electronic Patient Reporting Forms (PRF) as has been done in NHS Scotland [19], more patient-specific data including patient history and clinical observations such as pulse or blood pressure can provide further information about the patient at this step in the pathway. A further limitation is the lack of follow-up data for those patients who did not present to ED after GP referral; as such, we were unable to compare differences between this group and those who presented after GP referral or those who were directly conveyed. It is possible that patients who were referred to GP and did not present to hospital within 30 days could have died at home or experienced another adverse health event that was not captured at SRFT. Although the sample size was small, it was still able to detect statistically and clinically significantly results. The concern of multiple testing is small due to the exploratory nature of this pilot research. Thus, maintaining an alpha of 0.05, despite examining several associations in multiple cohort groups, enables hypotheses to be generated for future work while clinical recommendations based on these pilot findings are not appropriate at this stage.

This pilot study is the first to examine differences in patient outcomes between those directly conveyed to ED and those conveyed to alternative route of care; it supports the argument for further research to examine these differences to inform policy decisions. We are not aware of other studies that have examined differences in clinical outcomes between direct conveyance and GP referral. Despite limitations in data availability and a small sample size, this pilot study was still able to provide support for patient benefits associated with the GP AVS scheme. The scheme also gives clear benefits to both the patient and the wider healthcare system, as decreases in presentation at ED and decreases in admission rates may indicate an area for potential savings.

A large scale epidemiological retrospective cohort study, including a matched subject design on similar illness or injuries, would help identify patient characteristics associated with better and worse health outcomes as well as better practices of care (conveyance vs non-conveyance). The introduction of electronically available ambulance records could facilitate research in this area by indicating characteristics that predict ED attendance and admission and those that designate a GP referral as the optimal route of care. An economic analysis could examine the hypothesized savings associated with reducing unnecessary presentations at ED as well as unnecessary hospital admissions. Systematically understanding patient presentations, interventions and outcomes has the potential to create a data rich environment where clinical assessments and interventions to prevent admission can be evaluated with the aim that they be made available in the community or closer to the point of crisis.

## Conclusions

In conclusion, this pilot study has demonstrated that the GP AVS programme has the potential to safely decrease the number of unnecessary ED conveyances, identify the right place of care for the patient and improve emergency care pathways at the system level. If methodologically rigorous research can, at scale, demonstrate the benefits of alternative conveyance schemes and suggest evidence-based changes to the system of urgent and emergency care, the benefits for patients could be much greater by increasingly providing safer care closer to home.

## Data Availability

The data that support the findings of this study were provided by Salford Royal Foundation Trust but restrictions apply to the availability of these data, which were used under license for the current study, and so are not publicly available.

## Abbreviations

AVS: Acute Visiting Scheme
CADS: Computer Aided Dispatch System
EPR: Electronic Patient Records
ED: Emergency department
GP: General practitioner
HCP: Healthcare practitioner
MPDS: Medical Priority Dispatch System
NHS: National Health Service
NWAS: North West Ambulance Service
SRFT: Salford Royal NHS Foundation Trust
UK: United Kingdom
